# The Quality Sequencing Minimum (QSM): providing comprehensive, consistent, transparent next generation sequencing  data quality assurance

**DOI:** 10.12688/wellcomeopenres.14307.1

**Published:** 2018-04-04

**Authors:** Shazia Mahamdallie, Elise Ruark, Shawn Yost, Márton Münz, Anthony Renwick, Emma Poyastro-Pearson, Ann Strydom, Sheila Seal, Nazneen Rahman

**Affiliations:** 1 Division of Genetics & Epidemiology , The Institute of Cancer Research, London, SM2 5NG, UK; 2 TGLclinical, The Institute of Cancer Research, London, SM2 5NG, UK; 3 Cancer Genetics Unit, Royal Marsden NHS Foundation Trust, London, SM2 5PT, UK

**Keywords:** NGS, Quality Sequencing Minimum, quality assurance, quality control, depth of coverage, base quality, mapping quality, genetic testing

## Abstract

Next generation sequencing (NGS) is routinely used in clinical genetic testing. Quality management of NGS testing is essential to ensure performance is consistently and rigorously evaluated.

Three primary metrics are used in NGS quality evaluation: depth of coverage, base quality and mapping quality. To provide consistency and transparency in the utilisation of these metrics we present the Quality Sequencing Minimum (QSM).

The QSM defines the minimum quality requirement a laboratory has selected for depth of coverage (C), base quality (B) and mapping quality (M) and can be applied per base, exon, gene or other genomic region, as appropriate. The QSM format is CX_BY(P _Y_)_MZ(P _Z_). X is the parameter threshold for C, Y the parameter threshold for B, P _Y_ the percentage of reads that must reach Y, Z the parameter threshold for M, P _Z_ the percentage of reads that must reach Z. The data underlying the QSM is in the BAM file, so a QSM can be easily and automatically calculated in any NGS pipeline.

We used the QSM to optimise cancer predisposition gene testing using the TruSight Cancer Panel (TSCP). We set the QSM as C50_B10(85)_M20(95). Test regions falling below the QSM were automatically flagged for review, with 100/1471 test regions QSM-flagged in multiple individuals. Supplementing these regions with 132 additional probes improved performance in 85/100. We also used the QSM to optimise testing of genes with pseudogenes such as *PTEN* and *PMS2*. In TSCP data from 960 individuals the median number of regions that passed QSM per sample was 1429 (97%).  Importantly, the QSM can be used at an individual report level to provide succinct, comprehensive quality assurance information about individual test performance.

We believe many laboratories would find the QSM useful. Furthermore, widespread adoption of the QSM would facilitate consistent, transparent reporting of genetic test performance by different laboratories.

## Introduction

Next generation sequencing (NGS) is now routinely used to investigate if genomic variation has caused, or has the potential to cause human disease in clinical and research settings ^1^. Such genetic tests must robustly be able to detect pathogenic variants (positive tests) and to exclude the presence of pathogenic variants (negative tests). The accuracy of NGS in this regard is dependent on the performance of the assay generating the sequence data and the software tools that analyse the data. Suboptimal technical and/or analytical performance can lead to false positive or false negative results. Comprehensive quality management of NGS pipelines is thus essential and must encompass both quality assurance and quality control ^2^. Guidelines for quality management of the technical and analytical aspects of clinical NGS pipelines have been published ^2– 6^. One of the recommendations is for clear information about test performance and limitations to be provided on the test report, as healthcare professionals need this for clinical decision making ^3– 5^. Although this is well accepted to be best practice there are no specific guidelines for how it can be achieved, and most test reports provide limited or no information.

Three primary metrics are used to evaluate sequence quality in NGS data: depth of coverage (how many sequence reads are present at a given position), base quality (have the correct bases been called in sequence reads) and mapping quality (have the reads been mapped to the correct position in the genome) ^7^.

Insufficient depth of coverage is a common cause of false negative errors ^8^. A summary statement of coverage, such as the minimum depth or average depth of coverage achieved is often provided as a proxy for overall performance. However, using only the number of reads is not a sufficient measure of performance. False negative errors can still occur even with good depth of coverage, for example if the reads have been aligned incorrectly to the genome. Moreover, depth of coverage has limited utility in reducing false positive errors. Base quality and mapping quality are useful for this and most NGS base callers and read mappers provide Phred-scaled quality scores that quantify the probability that a particular base has been identified incorrectly (base quality score, BQ) ^9^, or a read has aligned to the wrong genomic position (mapping quality score, MQ) ^10^.

Quality assurance of an NGS test therefore requires attention to the quality control of depth of coverage, base quality and mapping quality during the design, optimisation and utilisation of the test. To bring consistency and transparency to these processes we have developed and implemented the Quality Sequencing Minimum (QSM). The QSM defines the minimum quality requirement that a laboratory has selected for depth of coverage (C), base quality (B) and mapping quality (M) and can be applied per base, exon, gene, or other genomic region, as appropriate. The QSM allows consistent, automated flagging of test regions that fall below minimum quality requirements and thus need additional scrutiny. In addition to its use in optimisation and quality control of NGS pipelines, the QSM can be used at an individual report level to provide succinct, comprehensive quality assurance information about individual test performance.

A standard BAM file contains the data required for the QSM and a QSM can be easily and automatically applied in any NGS pipeline. We have developed a freely available tool called CoverView to do this ^11^. Alternatively, custom scripting within any NGS analytical pipeline should readily allow application of a QSM. 

We have found use of a QSM a highly effective and efficient way to meet the quality management recommendations for NGS testing, and we believe others may also find it useful. Furthermore, general adoption of the QSM would help to standardise the communication of NGS quality information by the thousands of laboratories now undertaking NGS tests.

## Methods

A QSM includes three sequence quality metrics: depth of coverage (C), base quality (B), and mapping quality (M). The values for these metrics are dependent on the tools used to generate them. 

The standardised format for a fully comprehensive QSM is:

CX_BY(P _Y_)_MZ(P _Z_) using 1v1_2v2_..._nvn

Where X is the parameter threshold for C, Y is the parameter threshold for B, P _Y_ is the percentage of reads that must reach Y, Z is the parameter threshold for M, P _Z_ is the percentage of reads that must reach Z, and 1v1 through nvn are the tool name(s) and version(s) for read, base and mapping quality score generation, and variant calling.

Metric C uses the number of sequencing reads (depth of coverage) that have mapped to the reference genome for a given base position. Metric B uses the BQ of a base call that quantifies the probability that the base calling is incorrect. Metric M uses the MQ that quantifies the probability that a read is mapped to the wrong position during alignment.

The test region for which a QSM is generated is supplied in a BED file and the C, B, M values are in the sample’s BAM file and corresponding .BAI file. These are all routinely outputted by NGS analysis pipelines and used by the variant caller to detect and assign confidence to variant calls.

Variant callers apply data filtering at both the read-level and the base-level. Use of a preset parameter to exclude data is called ‘hard filtering’. If the variant caller performs hard filtering, these parameters should be used to define the minimum quality requirements for C, B and M. If the caller performs hard filtering at a read-level, but not at a base-level, the minimum quality requirement can be defined in a more flexible fashion.

The actual quality of NGS data generated may be well above a QSM, but any data of lower quality for any of the three metrics is automatically flagged for additional scrutiny. 

## Results

### Setting a QSM

TGLclinical is an ISO 15189 accredited clinical testing laboratory providing cancer predisposition gene testing using the TruSight Cancer Panel (TSCP) ^12^. TSCP uses Nextera® library preparation technology (Illumina, San Diego, CA, USA) and in-solution hybrid-selection chemistry for targeted enrichment. The TGLclinical analytical pipeline includes CASAVA v.1.8.2 to demultiplex and create FASTQs per sample from the raw base call (BCL) files and Stampy v.1.0.20 ^13^ with BWA v.0.7.5a pre-mapping ^14^ to map sequence reads to the human reference genome ( GRCh37). The pipeline uses Platypus v.0.2.4 ^15^ for variant calling of base substitutions and indels. 

The full QSM for TSCP testing in TGLclinical is:

C50_B10(85)_M20(95) using CASAVAv1.8.2_BWAv0.7.5a_Stampyv1.0.20_Platypusv0.2.4

This means that 100% of the constituent bases of the test region must minimally have a depth of coverage of ≥50 reads with a BQ of ≥10 in at least 85% of reads, and MQ of ≥20 in at least 95% of reads. The rationale for these choices is explained below.

For the QSM depth of coverage we selected C50, i.e. ≥50 reads per base. The average depth of coverage achieved for the TSCP pipeline in TGLclinical is >1000x. We consider a base position that fails to achieve 5% of the average depth of coverage (i.e. <50 reads) as suboptimal and requiring further evaluation. For many pipelines with much lower average depth of coverage, C50 would likely be too high, and would lead to an intolerably large number of additional evaluations.

For the QSM base quality minimum we selected B10(85), i.e. a BQ of ≥10 in at least 85% of reads. Platypus v.0.2.4, the variant caller used in TGLclinical, performs hard filtering at the read-level, discarding reads that have fewer than 20 bases with a quality score of BQ ≥20. Platypus uses retained reads to call variants, without hard filtering at the base-level, such that bases with BQ<20 in retained reads are used for calling variants. Bases of low quality tend to occur at the ends of reads ^16^, but to mitigate the impact of this, the TSCP probes are densely spaced giving read overlap. This means that a base can have a proportion of low (BQ<20) quality bases yet still have sufficient high quality bases for variant calling. To accommodate this we set the BQ minimum as 10 in at least 85% of reads.

For the QSM mapping quality minimum we selected M20(95), i.e. a MQ of ≥20 in at least 95% of reads. Platypus uses a mapping quality threshold of MQ ≥20 to perform hard filtering at the read-level. MQ is only generated per read so this hard filter also applies at the base-level. MQ is a reflection of the sequence context of the region. In a region with unique sequence context (i.e. not of low complexity or high homology with another part of the genome), only a small proportion of reads would have a low MQ by chance. We thus set the MQ minimum as 20 in at least 95% of reads.

### Using the QSM for optimisation and quality control of NGS pipelines

We have run TSCP in >20,000 samples and we have used the QSM in various ways to optimise TSCP testing as outlined below. Here we have specifically used TSCP data from 960 samples tested through the TGLclinical pipeline. The samples had been tested for one of two reasons: 1) The samples had undergone clinical diagnostic testing, the consent for which includes consent for quality assurance, audit and research. 2) The samples had been tested through our research studies to discover and characterise cancer predisposition genes, with written informed consent obtained. The research studies have been approved by the London Multicentre Research Ethics Committee (MREC/01/2/18, 05/MRE02/17, MREC/01/2/044) and Royal Marsden Research Ethics Committee (CCR1922).

As part of our automated NGS analysis pipeline sample BAM files with their corresponding .BAI index files are inputted into CoverView ^11^. We defined 1471 test regions in a BED file. Any test region falling below QSM C50_B10(85)_M20(95) for any of the three metrics was automatically flagged by CoverView in each sample (Dataset 1) ^17^.

To optimise TSCP we viewed each QSM-flagged region in the CoverView GUI by plotting the per base values for each quality metric C, B and M. Using the genomic coordinates of the TSCP 80-mer probes as a reference, we strategically designed additional probes across the QSM-flagged regions. In total we supplemented 100 test regions with 132 additional probes. This improved quality with 85/100 boosted regions meeting QSM C50_B10(85)_M20(95) ^17^. The booster probe set is now routinely analysed together with the original panel in all our tests. We call this the Optimised TSCP. 

Using the Optimised TSCP data for the 960 samples we outputted the number of samples in which the quality minimum for C, B, and/or M was not met, for each test region (Dataset 1) ^17^. Regions in which >1% of samples (i.e. ten or more samples) had not passed the quality minimum were reviewed. Across the 960 samples 1427/1471 (97%) test regions met C50, 1456/1471 (99%) met B10(85) and 1411/1471 (96%) met M20(95). Overall 1366/1471 (93%) test regions met QSM C50_B10(85)_M20(95). Per sample, the median number of regions that met QSM C50_B10(85)_M20(95) was 1429 (97%).

Detailed review of regions that were QSM-flagged in multiple individuals proved very useful for optimising TSCP performance. For example, some regions in genes with pseudogenes, such as *PTEN* and *PMS2*, were frequently flagged as not meeting M20(95). We evaluated these regions to see if the data was usable for variant calling. *PTEN* is located on chromosome 10q23 and has a pseudogene known as *PTENP1* on chromosome 9p13. The coding sequences of *PTEN* and *PTENP1* are identical apart from 18 dispersed bases (comparing ENST00000371953.3 for *PTEN* with ENST00000447117.1 for *PTENP1*), but the intronic sequences do not match. The high homology between *PTEN* and *PTENP1* leads to low mapping quality scores and five *PTEN* regions were flagged for not meeting the minimum requirement of M20(95) in the QSM evaluation data (Dataset 1) ^17^. To further evaluate *PTEN* testing performance with TSCP we reviewed data from 8133 samples. 47 different variants in 72 samples were detected, of which 18 were confirmed by Sanger sequencing and 29 were not confirmed ^17^. We also generated TSCP data in an additional 14 samples in which *PTEN* variants, in exons 2, 3, 5, 6, 7 and 9, had been identified by another method. All 14 were detected by TSCP. These data show that mapping quality below M20(95) does not compromise the detection of *PTEN* true positives and has a very low false positive rate of 0.4% (33/8133). We therefore do not routinely repeat *PTEN* regions that do not meet M20(95).

Our review of the QSM-flagged regions in *PMS2* led to a different conclusion. *PMS2* is on 7p22 and has a nearby pseudogene, called *PMS2CL*, that has high homology (>98%) for exons 12-15. All 960 samples failed M20(95) for exons 12-15 and the median proportion of reads with low mapping quality was 52–99% for these exons. To evaluate performance we used long-range PCR to avoid the pseudogene sequence, and evaluated 63 different variants detected amongst 4128 samples analysed by TSCP. 43/46 (93%) variants in exons 1-11 were confirmed. Only 10/17 (59%) variants in exons 12-15 were confirmed (Dataset 1) ^17^. We also reviewed the Sanger data for any additional variants that were not detected by TSCP and three variants, all in exon 14, were observed. Finally we generated TSCP data in five samples with *PMS2* variants, in exons 3, 4, 10 and 11, which had been detected by another method. All five were detected by the TSCP pipeline. Taken together these data show that TSCP performance for *PMS2* exons 1-11 is excellent, and fulfils the QSM. However, TSCP data for *PMS2* exons homologous to *PMS2CL* can lead to false negative and false positive results, and do not routinely fulfil the QSM. Therefore, if *PMS2* gene testing is requested we first perform TSCP, and if negative we perform Sanger sequencing with long-range PCR primers that avoid *PMS2CL*.

### Using a QSM on a gene test report

Detailed information about how the QSM was set and used to optimise testing would be provided in a laboratory’s full documentation and accreditation information. However, we believe the QSM also has potential utility at an individual report level. The QSM can succinctly provide consistent, transparent, comprehensive information about the performance of an individual test. This can be included on the individual test report and could be provided in different ways as shown in Table 1. Our personal preference is to include the short QSM statement ‘This test met QSM C50_B10(85)_M20(95)’. For tests in which some regions have not met QSM and were not repeated, for example because a pathogenic mutation was found in another gene, we would include a statement such as ‘This test met QSM C50_B10(85)_M20(95) {except *PMS2* exons 12-15}’. This provides clarity about the genes that have been fully or suboptimally tested.

**Table 1.** Potential QSM statements for inclusion on genetic test report.

QSM statement type	QSM met	TGLclinical report QSM statement example
Short QSM	Yes	This test met QSM C50_B10(85)_M20(95)
Short QSM	No	This test met QSM C50_B10(85)_M20(95) {except *PMS2* exons 12-15}
Full QSM	Yes	This test met QSM C50_B10(85)_M20(95) using CASAVAv1.8.2_ BWAv0.7.5a_Stampyv1.0.20_Platypusv0.2.4
Full QSM	No	This test met QSM C50_B10(85)_M20(95) using CASAVAv1.8.2_ BWAv0.7.5a_Stampyv1.0.20_Platypusv0.2.4 {except *PMS*2 exons 12-15}
Summary QSM	Yes	This test met QSM
Summary QSM	No	This test met QSM {except *PMS*2 exons 12-15}

## Conclusion

Quality assurance and quality control are essential requirements for genetic testing. It has proved challenging for laboratories to communicate how they are fulfilling these requirements for NGS tests. We have developed the Quality Sequencing Minimum (QSM), to achieve this. The QSM defines the minimum quality requirement a laboratory has selected for depth of coverage, base quality and mapping quality. The QSM is easy to generate and can be flexibly applied per base, exon, gene, or other genomic region, as best suits the laboratory. The QSM is very useful in the optimisation and quality control of NGS pipelines, allowing consistent, automated flagging of test regions that fall below the designated minimum quality requirements. The QSM can also be used at an individual report level to provide succinct, comprehensive quality assurance information about individual test performance. Widespread adoption of the QSM would facilitate consistent, transparent reporting of genetic test performance by different laboratories.

## Data availability

The below supporting data files have be archived as a single project file on open science framework: Dataset 1. Quality Sequencing Minimum - QSM http://doi.org/10.17605/OSF.IO/MY38Z under a CC0 1.0 Universal licence

Summary of QSM metrics and which were met in TSCP data from 960 samples

*PTEN* variants detected in 8133 samples analysed by TCSP and Sanger sequencing results

*PMS2* variants detected in 4128 samples analysed by TCSP and Sanger sequencing results

## Software availability

CoverView is available at: http://github.com/RahmanTeamDevelopment/CoverView/releases and www.icr.ac.uk/CoverView

CoverView documentation is available at: https://rahmanteamdevelopment.github.io/CoverView/

Latest source code: https://github.com/RahmanTeamDevelopment/CoverView

Archived source code as at time of publication: http://doi.org/10.5281/zenodo.1206100 ^18^

Software license: MIT License
