# Supplementary material for: The Quality Sequencing Minimum (QSM): providing comprehensive, consistent, transparent next generation sequencing  data quality assurance
Source: Wellcome Open Res. 2018 Apr 4;3:37. [Version 1] doi: 10.12688/wellcomeopenres.14307.1 (PMC6020721; doi:10.12688/wellcomeopenres.14307.1)
Supplement: Peer review history

# Peer review: Referee response for version 1

**DOI:** 10.21956/wellcomeopenres.15568.r33210

**Published:** 2018-06-26

**Authors:** Xiangtao Liu

**Affiliations:** 1 University of Iowa, Iowa City, IA, USA; 2 Tianjia Genomes LLC, Chaohu, Anhui, China

This paper presents a summary quality metric set QSM for NGS-based clinical assays, and its application to real clinical sequencing data collected from the cancer predisposition gene testing TGLclinical.

 While the three metrics contained in QSM are straightforward, the authors provided beneficial insights for panel-based clinical assays using real data. The evaluation on regions of genes with pseudogenes is nice work.

 The authors may extend to some work about effects of tumor purity, in additional to consideration on somatic mutations suggested by Erika.

I have read this submission. I believe that I have an appropriate level of expertise to confirm that it is of an acceptable scientific standard.

# Peer review: Referee response for version 1

**DOI:** 10.21956/wellcomeopenres.15568.r33208

**Published:** 2018-06-06

**Authors:** Ira M Lubin

**Affiliations:** 1 Primary lead of the Next-Generation Sequencing Standards for Clinical Testing, Centers for Disease Control and Prevention, Atlanta, GA, USA

Mahamdallie *et al *describe a much needed metric useful to the reporting of next-generation sequencing results.  There are a few elements within the manuscript requiring further clarification that are needed to support the validity of what is proposed.

 1. Page 3.  The paragraph beginning with "Variant callers apply data filtering at both...." Additional language will be useful in describing how this influences the QSM.  Variation in filtering can affect this score suggesting that some provision may be needed in describing how filtering is applied.

 2.  Page 3.  The paragraph beginning with "The actual quality of NGS data generated may be well above......" Related to this is the paragraph that begins on Page 4 beginning with "Detailed review of regions that were QSM-flagged........."  It is not clear when to know when to tag lower quality findings and if such findings occur, are the authors alluding to the idea that the QSM score does not apply in these cases.  Additional discussion regarding when the QSM is valid and not valid, what additional criteria should be reported to support these findings, in a general sense, should be described.  If all lower scores require further evaluation, this seems to defeat the intended simplicity of this measure.  In tagging low quality reads, does this indicate, sometimes, that the parameters set were too high?

 Page 4  Paragraph beginning with "For the QSM depth of coverage, we selected C50...."  Selection of coverage at C50 seems to be somewhat arbitrary.  It would be helpful if additional evidence or published studies can be cited.

 Page 4. Paragraph beginning with "For the QSM base quality minimum we selected B10(85)." and "For the QSM mapping quality minimum we selected M20(95)..."  The values chosen are rationalized in considering the filtering and analysis that Platypus provides.  There should be discussion regarding how this may vary using other methods.  This is somewhat addressed in Table 1 with the proposed format that includes the software used.  Additional discussion with the text would be helpful.  In reporting, one may also need to describe the application of QSM in conjunction with the types of variants targeted (e.g., SNPS, indels, repeats), each of which may have different requirements.  In addition allelic fraction may be important for cancer and mosaic samples.  This should be recognized within the text as a potential confounder.

I have read this submission. I believe that I have an appropriate level of expertise to confirm that it is of an acceptable scientific standard, however I have significant reservations, as outlined above.

# Peer review: Referee response for version 1

**DOI:** 10.21956/wellcomeopenres.15568.r32746

**Published:** 2018-04-16

**Authors:** Erika Souche

**Affiliations:** 1 Center for Human Genetics, University Hospitals Leuven, Leuven, Belgium

This paper describes a summary quality metric, Quality Sequencing Minimum (QSM), for Next Generation Sequencing based clinical assays. The proposed metric aims at assessing informative coverage in the region of interest as it only takes into account depth of coverage, base quality and mapping quality. The authors demonstrated the use of QSM during the development of a new assay where QSM allowed them to spot difficult to amplify/sequence regions. While some regions could be recovered by designing additional probes, some remained problematic. The authors thus checked whether their assay was able to properly genotype known mutations in difficult regions and adapted their diagnostic routing according to the results. Finally, the authors proposed to state QSM in the genetic test report.

 QSM can be a good alternative to genotype quality when using variant callers that do not compute genotype quality for reference calls. It would be interesting to compare genotype quality (from GATK for example) to QSM to see whether the same regions are flagged.

 While the QSM is generally well defined, it is difficult to understand why Py represents a proportion of reads and not a proportion of bases with a base quality above Y. Is one read included in the proportion if all its bases have a base quality above Y?

 In the introduction, "The QSM defines the minimum quality requirement that a laboratory has selected for depth of coverage (C), base quality (B) and mapping quality (M) and can be applied per base, exon, gene, or other genomic region, as appropriate", it should be clear that the QSM should be defined at the genetic test level, not at the laboratory level. For example the QSM will probably differ greatly between tests designed for somatic and germline mutations.

 Finally it would be nice to mention "informative coverage" and "region of interests (ROI)" as these are established concepts in clinical genetic testing.

I have read this submission. I believe that I have an appropriate level of expertise to confirm that it is of an acceptable scientific standard.
